# Development of monoclonal antibodies for quantification of bovine tumor necrosis factor-α

**DOI:** 10.3168/jdsc.2021-0123

**Published:** 2021-10-09

**Authors:** Anja Sipka, Susanna Babasyan, Sabine Mann, Heather Freer, Suzanne Klaessig, Bettina Wagner

**Affiliations:** Department for Population Medicine and Diagnostic Sciences, Cornell University, Ithaca, NY 14853

## Abstract

The expression of the proinflammatory cytokine tumor necrosis factor-α (TNF-α) is associated with production losses in dairy cows and is a hallmark of early inflammatory processes. Reliable tools for the detection and quantification of soluble as well as cytoplasmatic bovine TNF-α are needed to deepen our understanding of inflammatory dynamics in dairy cows. The objective of this study was to generate a monoclonal antibody (mAb) pair that could be used to quantify bovine TNF-α in cell culture supernatants and plasma and to detect cytoplasmatic TNF-α in bovine leukocyte populations. One mouse was immunized with a recombinant fusion protein of bovine TNF-α and equine IL-4 generated in Chinese hamster ovary cells. Murine monoclonal antibodies specific to bovine TNF-α were produced in hybridoma cell lines and selected based on their specificity to the recombinant IL-4/TNF-α protein. Clones 197-1 and 65-2, both murine IgG1 isotypes, detected the bovine TNF-α fusion protein as well as the native protein produced by peripheral blood mononuclear cells (PBMC) stimulated with a combination of phorbol myristate acetate and ionomycin. Both mAbs were tested for and lacked cross-reactivity to equine IL-4 and 3 other recombinant bovine cytokines (IFN-γ, IL-10, and CCL5) and were used to develop a fluorescent bead-based assay. The range of bovine TNF-α detection in the assay was 0.2 to 620 ng/mL, and the test was used to quantify native bovine TNF-α in cell culture supernatants of stimulated PBMC and in plasma from ex vivo whole-blood stimulations. Sample matrices were spiked with TNF-α, with subsequent recovery rates (mean ± SD) of 89% ± 9 (n = 3) in culture medium and 94% ± 12 (n = 3) in heat-inactivated fetal bovine serum. Serial dilutions of plasma and cell culture supernatants from stimulated whole blood or PBMC indicated excellent accuracy for quantification of native TNF-α in bovine samples. Both bovine TNF-α mAbs also detected intracellular TNF-α in bovine CD14^+^ monocytes and CD4^+^/CD8^+^ lymphocytes. In conclusion, we demonstrated that the mAbs generated provide valuable new tools to quantify native bovine TNF-α in a wide concentration range and to characterize intracellular TNF-α expression in bovine leukocytes.

## Highlights

• Tumor necrosis factor-α (TNF-α) is a key mediator of early inflammation in dairy cows.
• We generated 2 monoclonal antibodies specific to bovine TNF-α.
• These monoclonal antibodies can be used to quantify soluble TNF-α in a bead-based assay.
• In flow cytometric application, they can differentiate cytoplasmatic TNF-α in leukocytes.

## Graphical Abstract

**Figure:**
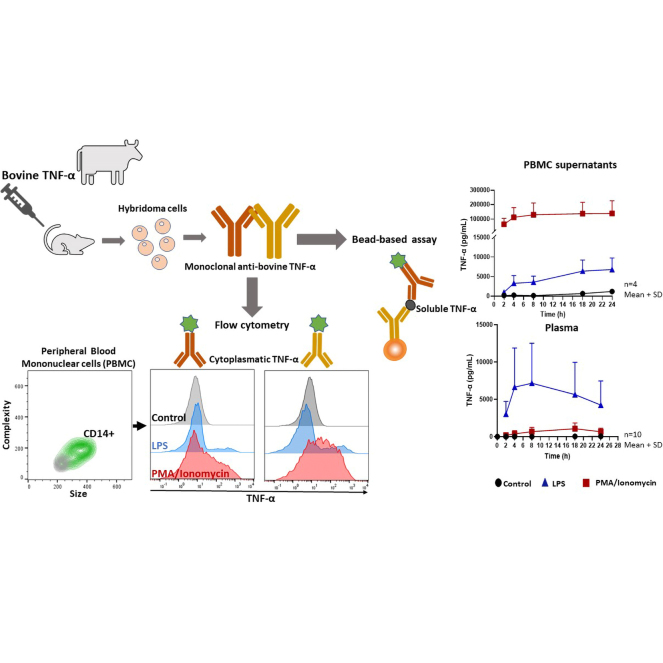
**Summary:** We generated a monoclonal antibody (mAb) pair specific to bovine tumor necrosis factor-α (TNF-α) by immunizing one mouse with a recombinant fusion protein of bovine TNF-α and equine IL-4 generated in mammalian cells. Two murine IgG1 mAbs specific to bovine TNF-α were produced in hybridoma cell lines and selected based on their specificity to the recombinant IL-4/TNF-α protein and the native protein produced by peripheral blood mononuclear cells (PBMC). Both mAbs lacked cross-reactivity to equine IL-4 and 3 other recombinant bovine cytokines. A bead-based assay was developed and used to quantify native bovine TNF-α in PBMC culture supernatants and in plasma from ex vivo whole-blood stimulations. In a flow cytometric assay, both mAbs detected intracellular TNF-α in bovine CD14^+^ monocytes and CD4^+^/CD8^+^ lymphocytes. Our bovine TNF-α mAb clones specifically and accurately quantified soluble TNF-α in cell culture supernatants and plasma and detected cytoplasmatic TNF-α in bovine leukocytes.

Tumor necrosis factor-α (**TNF-α**) is an important proinflammatory cytokine characterizing the early inflammatory response (Kany et al., 2019). In dairy cows, increased levels of TNF-α have been associated with immune dysfunction and reduced milk production (Sordillo et al., 1995; Yuan et al., 2013). To deepen our understanding of these associations, a reliable method is needed to determine concentrations of key inflammatory mediators such as TNF-α in bodily fluids as well as their cytoplasmatic expression in leukocyte populations. Monoclonal antibodies (mAb) against bovine TNF-α are available, but data on their sensitivity and specificity in different bovine samples are limited. Moreover, their ability to detect native protein in plasma has not been described (Paape et al., 2002; Kwong et al., 2010). Our objective was to generate novel mAbs to quantify bovine TNF-α in cell culture supernatants and plasma using a bead-based assay and to enable flow-cytometry–based detection of cytoplasmatic TNF-α in bovine peripheral blood cells.

The procedures performed on mice were approved by the institutional animal care and use committee (IACUC) at Cornell University (IACUC protocol 2007–0079). Sampling procedures in cows were approved under IACUC protocol number 2017–0107.

Monoclonal antibodies specific to bovine TNF-α were produced as previously described for equine proteins (Wagner et al., 2012). Briefly, the coding sequence (cDNA) for soluble bovine TNF-α (GenBank Accession NM_173966.3, bases 194–898) was amplified from bovine peripheral blood mononuclear cells (**PBMC**) using the forward primer AAGGATCCCAGGTCCTCTTCTCAAGCCTCAAGTAAC, including a *Bam*HI restriction site (underlined), and the reverse primer ATATATAAGCTTTCACAGGGCGATGATCCCAAAGTAGACC, including a *Hin*dIII restriction site (underlined), and cloned into the mammalian IL-4 or IgG1 fusion protein expression vectors. Subsequently, TNF-α was expressed as a fusion protein with equine IL-4 or IgG1 in Chinese hamster ovary (**CHO**) cell lines as previously described (Wagner et al., 2005). The nucleotide sequence of the cloned TNF-α was identical to the reference sequence (NM_173966.3). For immunization, the recombinant fusion protein IL-4/TNF-α was purified from the growth medium by affinity chromatography using an anti-IL-4 coupled NHS (*N*-hydroxysuccinimide)-activated Sepharose column (Millipore Sigma) as previously described (Wagner et al., 2012). Immunization of a BALB/c mouse and the fusion procedure to produce hybridoma cell lines were performed as previously described (Wagner et al., 2008). Hybridoma cell lines expressing antibodies against bovine TNF-α were selected by ELISA. For ELISA, the plates were either coated with 0.7 µg/mL goat anti-horse IL-4 polyclonal antibody (Novus Biologicals) followed by incubation with supernatant from CHO cells expressing the IL-4/TNF-α fusion protein, or incubated directly with purified IL-4/TNF-α fusion protein at 1 µg/mL. In both assays, the supernatants from hybridoma clones were then used to detect the antigen followed by horseradish peroxidase (HRP)-conjugated goat anti-mouse IgG polyclonal antibody (Jackson Immuno Research Labs). Hybridoma clones were further selected by flow cytometry using transiently transfected CHO cells expressing TNF-α/IgG1 fusion proteins. Transiently transfected CHO cells were fixed with 2% paraformaldehyde and labeled intracellularly with hybridoma supernatants in saponin buffer (PBS, 0.5% BSA, 0.5% saponin, 0.02% NaN_3_) followed by labeling with A647-conjugated goat anti-mouse IgG polyclonal antibody Fab_2_ fragment (ThermoFisher Scientific). For specificity testing of each clone, ExpiCHO cells transfected with IL-4 and an irrelevant protein were used. Hybridoma clones that detected TNF-α were expanded and purified using Protein G affinity columns (GE Healthcare). Protein concentrations were determined by bicinchoninic acid assay (Pierce BCA protein assay kit, Thermo Scientific). The murine isotypes of these hybridoma clones were determined using mouse monoclonal antibody isotyping reagents (Millipore Sigma).

Potential cross-reactivity of the mAbs with other bovine cytokines was tested by flow cytometry against 3 recombinant bovine fusion proteins (IL-10/IgG1, IFN-γ/IgG1, and CCL5/IgG1). Chinese hamster ovary cells transfected with the respective bovine fusion protein constructs or TNF-α/IgG1 as positive control were fixed with 2% paraformaldehyde and intracellularly labeled with the purified hybridoma clones as well as with isotype controls for mouse IgG1. Subsequently, cells were labeled with a fluorescein isothiocyanate (FITC)-conjugated goat anti-mouse polyclonal antibody.

Detection of native bovine TNF-α was tested in bovine whole blood and bovine PBMC. Blood samples were obtained from adult, lactating cows by venipuncture of the coccygeal vessels, and PBMC were separated by density gradient centrifugation as previously described (Sipka et al., 2016).

Bovine PBMC were stimulated with *Escherichia coli* LPS (O111:B4, 100 ng/mL) or a mix of phorbol myristate acetate (**PMA**, 25 ng/mL) and ionomycin (750 ng/mL, all from Millipore Sigma) for 4 h at 37°C and 5% CO_2_ enriched atmosphere or left as an unstimulated control. All cultures contained brefeldin A (0.5 μg/mL, Millipore Sigma) to prevent exocytosis of the native protein. After a 4-h incubation, cells were fixed with 2% paraformaldehyde. Cells were labeled in saponin buffer with purified, biotinylated mAbs and in parallel with mAbs against bovine CD14 conjugated with FITC (clone CC-G33, BioRad) and a mixture of anti-bovine CD4 and CD8 mAbs both conjugated with A647 (clones CC-8 and CC-63, BioRad). Subsequently, cells were labeled with streptavidin R-phycoerythrin (ThermoFisher Scientific). Isotype-matched control antibodies were included in all experiments [mouse IgG1 (MCA 1209) and IgG2a (MCA 1210) negative controls, BioRad]. All samples were analyzed with a FACS Canto II flow cytometer (BD Biosciences). For CHO cells, 10,000 events per sample, and for PBMC, 50,000 events per sample were acquired. All cells were gated as singlets according to their FSC height and area. The CHO cells were analyzed as a homogeneous group, whereas PBMC were gated by their expression of CD4/CD8 (T lymphocytes) and CD14 (monocyte subset).

For the development of a fluorescent bead-based assay, purified mAbs were tested to identify pairs of coupling and detection antibodies using a TNF-α/IgG1 fusion protein standard (Wagner and Freer, 2009). The TNF-α/IgG1 fusion protein was derived from supernatants of CHO cell transfectants as described above. Standard concentration was determined by quantification of the IgG1 portion of the equine IL-4/IgG1 fusion protein with an IgG1 protein standard of known concentration (Wagner et al., 2005). An aliquot of each mAb was coupled to fluorescent beads (MicroPlex Microspheres, Luminex) and paired in the assay with one or the other biotinylated mAb (EZ-Link Sulfo-NHS-Bioton, ThermoFisher Scientific) as detection antibody. The beads were incubated with a dilution series of the TNF-α standard or samples followed by incubation with the biotinylated detection mAbs and streptavidin R-phycoerythrin (ThermoFisher Scientific). Samples were read in a BioPlex 200 instrument (BioRad) and concentrations expressed in picograms per milliliter. The final assay (mAb 197-1 for bead coupling and biotinylated mAb 65-2 for detection) was then used to determine the assay's linear quantification range for TNF-α in serial dilutions of cell culture supernatants from PBMC stimulated with PMA/ionomycin, or plasma from whole blood after stimulation with LPS (100 mg/mL). Specificity of the assay for TNF-α was confirmed by testing both mAbs against IgG1 fusion protein standards of recombinant bovine IFN-γ, IL-10, and CCL5 in the bead-based assay.

Plasma samples were obtained from in vitro stimulation of whole-blood samples from 10 adult lactating cows. Whole blood was incubated with a mix of PMA/ionomycin or LPS (see above) or left unstimulated (control) for 2, 4, 8, 18, and 24 h at 38°C. Plasma was harvested by centrifugation for 10 min at 1,500 × *g* and 4°C. In parallel, PBMC from 4 adult lactating cows were cultured at 37°C in a 5% CO_2_ enriched atmosphere, applying the same treatments and time points as for the whole-blood stimulation. Cell culture supernatants were collected by centrifugation for 10 min at 250 × *g* and 4°C. Samples were stored at −80°C until analyzed.

In the initial screening of the hybridoma supernatants, mAb clones 65-2 and 197-1 detected the IL-4/TNF-α fusion protein by ELISA and flow cytometry (data not shown). The TNF-α mAb clones were expanded, purified, and further characterized by intracellular staining of different CHO cell transfectants followed by flow cytometry to both be of the IgG1 isotype. They did specifically detect transfectants expressing TNF-α/IgG1 while not reacting with transfectants of 3 other recombinant bovine proteins (IL-10/IgG1, IFN-γ/IgG1, and CCL5/IgG1) or the equine recombinant IL-4/IgG1 fusion protein, indicating specific binding to recombinant bovine TNF-α and not the equine IgG1 portion of the fusion protein or the other bovine cytokines tested (Figure 1).

**Figure 1:**
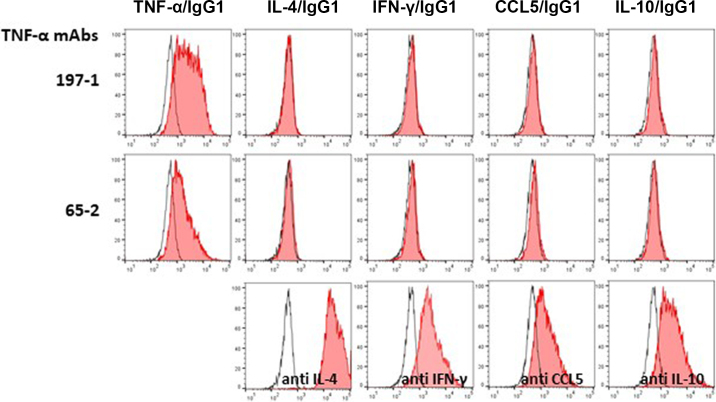
Reactivity of the newly developed monoclonal antibodies (mAb) 197-1 and 65-2 against bovine tumor necrosis factor-α (TNF-α) with Chinese hamster ovary cell transfectants expressing different bovine cytokines. Both mAb clones were tested for detection of TNF-α fusion protein and for cross reactivity with other bovine IgG1 fusion proteins (IFN-γ, IL-10, and CCL5) and a recombinant equine IL-4/IgG1 fusion protein. Chinese hamster ovary cells were transfected with the respective bovine fusion proteins or TNF-α/IgG1. Cells were fixed with 2% paraformaldehyde and intracellularly labeled with the TNF-α mAbs or specific mAbs for the other cytokines or IgG1 (filled histograms), as well as with isotype controls for mouse IgG1 (open histograms). Subsequently cells were labeled with a fluorescein isothiocyanate (FITC)-conjugated goat anti-mouse polyclonal antibody.

Subsequently, TNF-α clones 197-1 and 65-2 were used to develop a bead-based assay for the detection of soluble TNF-α in PBMC culture supernatants and plasma. Each mAb clone was evaluated as a bead-coupled capture antibody and as a biotinylated detection antibody. We did not perform epitope mapping, but the results from pairing both mAb in the bead-based assay indicated that they recognize different epitopes of TNF-α. The use of clone 197-1 as capture antibody and 65-2 as detection antibody resulted in detection of bovine recombinant TNF-α/IgG1 fusion protein (620 ng/mL) with a high mean fluorescence intensity (**MFI**) of 17,785 ± 898 (n = 11), as well as native protein in culture supernatants of PBMC stimulated with a mix of PMA and ionomycin (MFI: 11,681 ± 1,680, n = 4) and low blank values (MFI: 3 ± 0.2, n = 11). The range of TNF-α detection for the assay was 0.2 to 620 ng/mL (Figure 2A). This is comparable to the range for a bead-based bovine TNF-α assay described by others (Dernfalk et al., 2007; Farney et al., 2011), who used a polyclonal antibody for detection. To monitor consistent performance of the assay, we produced a Levey-Jennings chart (data not shown) applying a control limit of the mean ± 2 standard deviations (Carroll et al., 2003). All assays performed were within control limits. Both intra- and interassay CV were 5% (calculated from 4 replicates per plate on 3 plates of cell culture supernatants from PBMC stimulated with a mix of PMA and ionomycin). Accuracy of detection was determined by recovery rate from sample matrices spiked with the fusion protein and linearity of serial dilutions of the fusion protein standard, PBMC supernatants, and plasma samples. Recovery rate of the fusion protein from fetal bovine serum (FBS) was 89% ± 9 and from cell culture medium (RPMI-1640 + 5% FBS) was 94% ± 12. Serial dilutions of fusion protein standard and cell culture supernatants and plasma from stimulated PBMC or whole blood were parallel (Figure 2B), demonstrating that the native protein was detected accurately across tested sample matrices.

**Figure 2:**
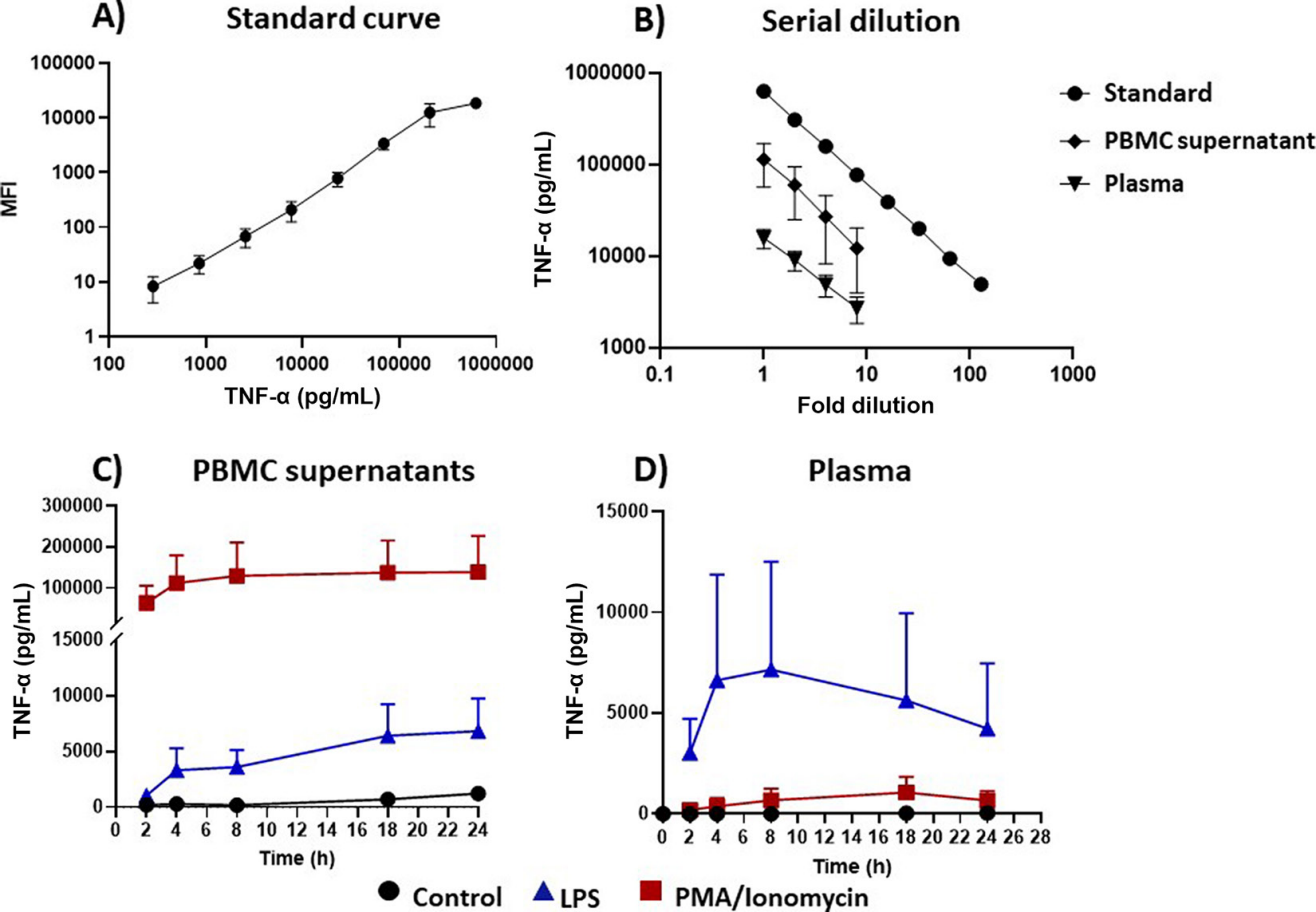
Quantification of bovine tumor necrosis factor-α (TNF-α) in cell culture supernatants and plasma. Bovine TNF-α in bovine peripheral blood mononuclear cell (PBMC) culture supernatants and plasma was detected with a fluorescent-bead based assay using anti-bovine TNF-α clone 197-1 as bead-coupled capture antibody and clone 65-2 as biotinylated detection antibody. Whole blood and PBMC were stimulated with LPS (100 ng/mL) or a mix of phorbol myristate acetate (PMA, 25 ng/mL) and ionomycin (750 ng/mL) or left unstimulated (control). (A) Serial dilution of a TNF-α fusion protein derived from supernatants of a Chinese hamster ovary cell transfectant was used as standard curve. Data are shown as mean fluorescence intensity (MFI) plus standard deviation of 8 assays. (B) Serial dilutions of the TNF-α standard, PMA/ionomycin-stimulated PBMC culture supernatants, and plasma from LPS-stimulated whole blood were analyzed in parallel. (C) PBMC (n = 4) or (D) whole-blood samples (n = 10) were stimulated for 2, 4, 8, 18, or 24 h. Data are shown as mean ± SD.

The mAb pair was then used to detect native TNF-α in PBMC cell culture supernatants from 4 different cows, and plasma samples from 10 blood samples for whole-blood stimulation at 5 time points over 24 h (Figure 2C, 2D). An approximately 10-fold higher concentration of TNF-α was found in supernatants from PMA/ionomycin-stimulated PBMC compared with supernatants from LPS-stimulated cells (Figure 2C). This result was expected because LPS mainly induces TNF-α production in monocytes and macrophages, whereas the combination of PMA and ionomycin is a strong inducer of cytokine production across all leukocyte types (Rossol et al., 2011). In plasma from whole-blood stimulation, however, LPS induced a much higher concentration of TNF-α, and PMA/ionomycin-stimulated samples showed low to no detectable TNF-α (Figure 2D). Dandrieux et al. (2019) observed similar results after stimulation of canine whole blood with lower TNF-α concentrations in response to PMA/ionomycin compared with LPS stimulation. The discrepancy between the response to PMA/ionomycin in PBMC and in whole blood can be associated with the difference in cell composition in the 2 samples. Whereas the PBMC represent a highly purified fraction of mononuclear leukocytes, whole blood contains many other cell types, such as neutrophils and large numbers of red blood cells and platelets. The calcium ionophore ionomycin and the protein kinase C activator PMA stimulate a broad spectrum of cells, including platelets (Watanabe et al., 2001), lymphocytes (Moss et al., 2000; Crawford et al., 2014), monocytes (Schnabel et al., 2018), neutrophils (Lundqvist-Gustafsson and Bengtsson, 1999), and endothelial cells (Jia et al., 2013). Thus, if PBMC and whole blood cells are stimulated with the same concentration of PMA/ionomycin, the effect in whole blood is distributed across all blood cells based on their proportion in the sample. Therefore, less of the stimulant is available to activate mononuclear cells in the sample to produce TNF-α. In contrast, PBMC stimulation directly targets the most effective TNF-α–producing cells. Stimulation with LPS, on the other hand, specifically targets monocytes in both whole blood and PBMC, through the LPS receptor that is primarily expressed by this cell type, leading to a TNF-α response in a similar concentration range in both samples (Paape et al., 1996; Guo et al., 2009). However, the variation between individual samples was much higher in plasma than in PBMC supernatants. High animal variation in plasma levels of TNF-α in cattle in response to LPS has been described by others (Sacchini et al., 2012; Cousillas-Boam et al., 2020). Several factors can influence variation in cytokine response. Differences in monocyte counts could influence TNF-α concentration in ex vivo whole-blood stimulation. In this study, we did not obtain leukocyte differential counts, but for investigations with the goal of describing inflammatory profiles, information on leukocyte composition is recommended. The time course showed that concentrations of TNF-α in PBMC supernatants reached a plateau after 4 h of stimulation with PMA/ionomycin and stayed constant for the remainder of the time. Stimulation with LPS resulted in a maximum response after 18 h, with comparable values detectable after 24 h. In contrast, plasma samples showed highest concentrations of TNF-α at 4 and 8 h following LPS stimulation and a decline at 18 and 24 h. This experiment indicates that for whole-blood stimulation, a shorter incubation time of 4 h and the use of leukocyte-specific stimulation is advisable to capture the maximum TNF-α response.

Both mAbs were used in intracellular labeling to test for presence of cytoplasmatic TNF-α in bovine PBMC populations (Figure 3A, 3B). After 4 h of stimulation with either PMA/ionomycin or LPS, differential expression of cytoplasmatic TNF-α could be detected in PBMC, whereas TNF-α was not detected in untreated PBMC (Figure 3A). We then explored TNF-α expression in different PBMC populations. Stimulation with LPS induced TNF-α expression only in a subset of CD14^+^ monocytes (Figure 3B). Bovine monocytes are a heterogeneous cell population characterized by surface expression levels of CD14 and CD16 as classical, intermediate, and nonclassical monocytes, with differences in their inflammatory response (Hussen et al., 2013). In this experiment, we focused on surface expression of CD14 but did not investigate CD16 expression. Our results likely reflect the proportion of different subsets in PBMC, with some producing more TNF-α than others in response to stimulation with LPS. The lack of TNF-α expression in response to LPS in CD4^+^/CD8^+^ lymphocytes was expected because this cell population typically does not express LPS receptors (Howard et al., 1996). Stimulation with the more generic cell activator PMA/ionomycin induced a robust TNF-α response in CD14^+^ monocytes and in CD4^+^/CD8^+^ lymphocytes (Figure 3B), which is in line with previous reports (Bueno et al., 2002; Cepika et al., 2010). Our objective was to demonstrate that the newly developed antibodies can detect intracellular TNF-α in stimulated PBMC. Our results from flow cytometric analyses of cytoplasmatic TNF-α in PBMC populations complement the quantification of TNF-α in PBMC cell culture supernatants (Figure 2C). It should be noted that we did not explore TNF-α production during responsive and refractory states of different leukocyte types. The developed antibody pair could be used in future experiments that aim to describe TNF-α production in different cell types over time.

**Figure 3:**
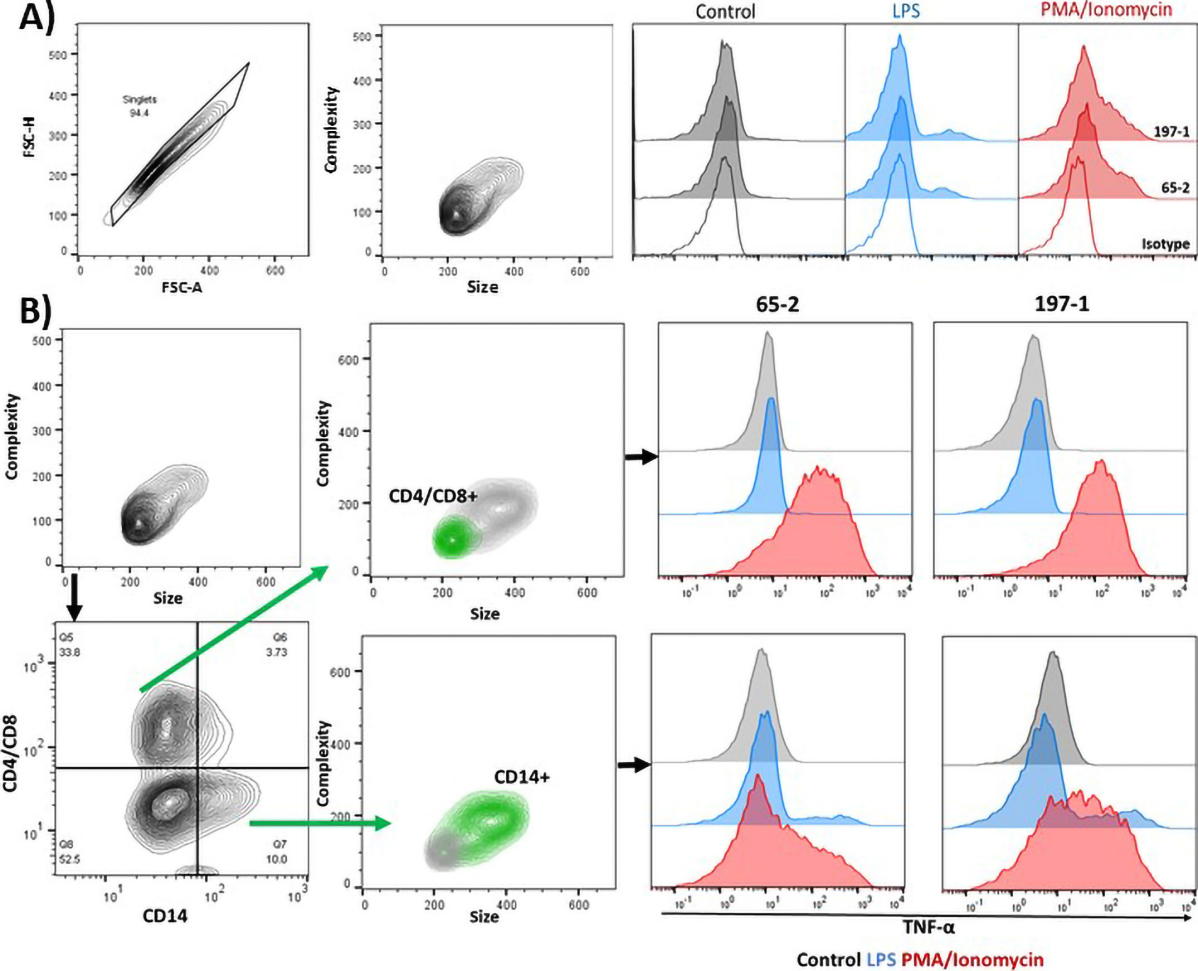
Detection of intracellular tumor necrosis factor-α (TNF-α) in bovine peripheral blood mononuclear cells (PBMC) by flow cytometric analysis. Cells were stimulated for 4 h with LPS (100 ng/mL) or a mix of phorbol myristate acetate (PMA, 25 ng/mL) and ionomycin (750 ng/mL) or left unstimulated (control). All cultures contained brefeldin A (0.5 μg/mL). (A) Contour plots and histograms were gated on single events based on forward scatter height (FSC-H) and area (FSC-A). Fixed cells were labeled intracellularly with biotinylated anti-bovine TNF-α clones 65-2 or 197-1 followed by streptavidin R-phycoerythrin or with an isotype control to test for unspecific binding of mouse IgG1. (B) Furthermore, cells were labeled with anti-bovine CD14-FITC (fluorescein isothiocyanate; clone CC-G33) and a mix of anti-bovine CD4-A647 (clone CC8) and anti-bovine CD8-A647 (clone CC63) to assess TNF-α production in CD14^+^ monocytes and CD4^+^/CD8^+^ lymphocytes. Graphs are representative of results from 4 animals.

In conclusion, the new bovine TNF-α mAb clones 65-2 and 197-1 specifically and accurately quantified soluble TNF-α in cell culture supernatants and plasma samples in a bead-based assay across a wide concentration range. In addition, both mAbs could differentiate cytoplasmatic TNF-α expression in bovine PBMC populations by flow cytometry. The newly developed mAb pair can be used as a tool to describe inflammatory responses in dairy cows in a variety of ex vivo– and in vitro–derived samples. Last, ideal stimulation times and stimuli concentrations for optimal TNF-α measurements can vary for whole blood and PBMC.
